# Cosmetic and Application Qualities of the Cream Physiorelax Compared to Another Topical Cream for Musculoskeletal Health Care: An Organoleptic Expert Panel Approach

**DOI:** 10.1111/jocd.70368

**Published:** 2025-09-22

**Authors:** Jordi Bertrán Novella, Nuria Garcia Gonzalez, Pere Guiró Coll, David Asensio Torres, Sonia Palenzuela Larrarte, Mónica Giménez

**Affiliations:** ^1^ Sports Specialist Physiotherapist Barcelona Spain; ^2^ Almirall R&D, Sant Feliu De Llobregat Barcelona Spain; ^3^ Almirall HQ MCI Barcelona Spain; ^4^ Medical Writing Department TFS HealthScience Barcelona Spain

**Keywords:** formulation/stability, muscular fatigue, natural products, personal care products, safety testing, skin barrier

## Abstract

**Objective:**

Regular massages can be an instrument to help the body recover and maintain health. The growing trend toward musculoskeletal personal health care is accompanied by the use of natural products. When deciding between topical products, their characteristics become crucial, especially in sensitive skin where treatments are applied routinely. This study evaluates for the first time the cosmetic and application properties of Physiorelax, a natural musculoskeletal health care product suitable for muscle and ligament massage, compared to Fisiocrem.

**Methods:**

This is a sensory panel methodology, using a mixed descriptive approach (qualitative/quantitative). Twenty panelists trained in quantitative sensory analysis since 2018 evaluated the two products. Product presentation was randomized and blinded. Cosmetic results are presented as percent agreement among panelists and application aspects as quantitative scores.

**Results:**

Both products show a comparable sensory profile, in terms of application properties: shape integrity, firmness, moisture/grease balance, spreadability, low tack afterfeel, and residue. However, in terms of an overall preference for product, 90% of panelists liked Pshysiorelax, against 70% for Fisiocrem. An additional 10% of panelists disliked Fisiocrem over Pshysiorelax (chi‐square = 12.5, *p* = 0.002). The results should be interpreted considering the inherent limitations of the panel design itself. Future studies could assess the preference of use of the product by daily care professionals and the general population.

**Conclusion:**

Considering the panelists' general preference for Physiorelax, and the addition in this product of extra natural ingredients preserving skin care, Physiorelax seems a very good candidate for daily use for musculoskeletal health care and as a massage cream for muscles and ligaments in personal care.

## 1 Introduction

According to the World Health Organization (WHO), musculoskeletal conditions are relevant across the life‐course—from childhood to older age [1]. Musculoskeletal pain affects approximately 47% of the general population [2], and is associated with physical and mental health problems in most cases [3, 4], leading to a significant socioeconomic impact [5]. Thus, maintaining locomotor health and preventing early problems and discomfort is fundamental during adult life [6].

For the treatment not only of musculoskeletal pain, regardless of its origin, but also for muscle and ligament conditioning and maintenance, more and more health professionals dedicated to this field (e.g., physiotherapists) consider that the use of analgesics and anti‐inflammatory drugs should be reduced. Because of the significant side effect profiles and interaction effects of some pharmaceutical drugs [7], there is a greater interest in natural remedies and cosmetic products, such as those containing herbs, extracts, plants, and oil essences [2, 8, 9, 10].

In recent years, there has been a growing concern for musculoskeletal health [11], a need for self‐care that goes hand in hand with an increase in natural self‐care. This trend is likely to continue, creating opportunities for natural ingredients used in herbal healthy products. Precisely, one of the most common conditions for which adults use herbal and natural medicines is pain and musculoskeletal health [12]. In this sense, plants and natural skin‐friendly topical products may be a good alternative especially when used in persons with some chronic skin sensitive disorders (e.g., psoriasis) [13], where skin care is a very important conditioning factor to bear in mind, and they do not present interactions with other drugs.

Natural products, and their possibilities, are currently a matter of great interest in the pharmaceutical and cosmetics industries [14, 15, 16], with a number of scientific studies in galenic pharmacy increasing rapidly [17]. Within this scenario, it is important that the products for personal care and health preservation contain the preferences and motivational characteristics that they claim to maintain the continuity of treatment use. For example, one of the main motivations for using natural cosmetic products in personal care in a group of Spanish women (2022) was precisely the healthy character of the product and the good results on skin (answered by the 55% and 48% of the sample, respectively) (Main reasons for using natural beauty products Spain 2022 | Statista). In addition, in most cases health professionals are also involved in the maintenance of the musculoskeletal health of people, so they must be in accordance with what the product offers to them in sensory terms, as they are also in direct contact with the product. Therefore, it is important to know the organoleptic characteristics of the natural products that may be in use, both for the person who directly uses them and for the health professional who can also apply them in their daily practice [18].

Among the creams for daily personal use for musculoskeletal health care, including maintenance of muscles and ligaments, is Physiorelax (https://www.physiorelaxforte.com/). This product is based on vegetable extracts with a base of helenanin2 [19, 20, 21] that contains four high‐quality vegetable oils (arnica, *harpagophytum*, *hypericum* and calendula) in a single complex, which achieves a high homogeneity of the formula, and which can be used on sensitive skin. Complementarily, it also contains natural extracts designed for professional hand care.

To our knowledge, there is not an organoleptic description of this personal care product. Therefore, the present study aims to evaluate the cosmetic and characteristics of application of Physiorelax, using a descriptive panel of experts in sensory analysis of topical products, and comparing this product with Fisiocrem, another cosmetic for the maintenance of muscles and ligaments. The choice of this comparative is mainly because both products contain natural ingredients and are designed for the care and maintenance of musculoskeletal health, which makes them directly comparable in terms of application and efficacy. This comparison could provide a valuable benchmark to understand the positioning of Physiorelax in relation to other products on the market, in terms of quality and user preference. The results drawn from this panel of experts may facilitate, then, the choice of a product by health professionals involved in muscle and ligament massage.

## 2 Methods

### 2.1 Panel Selection and Training

The present descriptive sensory study is based on a panel of 20 independent experts in sensory analysis of topical products (6 men/14 women, aged 36–59 years) from three Almirall company units in Barcelona (Spain) to assess, by a mixed method (qualitative and quantitative), the cosmetic and application characteristics of the galenic cream product Physiorelax, compared to Fisiocrem, as well as their overall product rating.

The selection of panelists (or assessors) was based on the criteria and questionnaires of the American Society for Testing and Materials (ASTM) international guidance [22]. The assessors were selected from within Almirall company. Candidates were contacted by interoffice memorandum, email, company newsletter, or notices posted on regular and electronic noticeboards. Prior to the prescreening questionnaire, candidates were informed of the time spent on training, the potential duration of the panel, the use of the panel, and the expectations of each assessor in relation to the panel's responsibilities. Candidates participated in the tests in two separate sessions. For this panel of 20 final evaluators, 500 candidates from all over the company applied, of which 170 candidates remained in the second selection phase on the basis of a preselection questionnaire (ASTM guidelines) [22]. The expert panelists were chosen through a blind selection process. The final panel was composed of chemists, pharmacists, biologists, information technology specialists and analysts, among others. The expert panel recruited for the study has been participating in annual dermatological training since 2018.

The sensory panel study was conducted internally. As these are studies in which no active ingredients are used, no institutional or research ethics approval was required. For more information on the selection and training of panelists, see the ASTM guidelines [22].

### 2.2 Products for the Sensory Evaluation

Both Physiorelax (Physiorelax Forte Plus Cream, Almirall) and Fisiocrem (Fisiocrem Active Gel, Uriach) are natural topical products for use in the preparation, protection, and recovery of muscles, joints, and ligaments, before or after any activity of daily life or physical exercise. However, their use has been extended due to their effects after blows, contractures, or sprains, also helping the reabsorption of oedemas and haematomas. While Physiorelax Forte Plus Cream has been considered by the Spanish Agency of Medicines and Health Products (AEMPs) as a personal care product, Fisiocrem Active Gel is classified as a cosmetic.

Physiorelax Forte Plus is made up of 90% natural ingredients (https://www.physiorelaxforte.com/). The formula includes, among its natural ingredients, flower extract of arnica ( *Arnica Montana* ), harpagophytum ( *Harpagophytum Procumbens* ) root extract, calendula ( *Calendula Officinalis* ) flower extract, hypericum ( *Hypericum Perforatum* ) flower/leaf extract, mentha (*Mentha Piperita*), menthol, camphor, *Helianthus Annuus* seed oil, rosa moschata seed oil, glycine soja oil, and thermal water. It also includes limonene, citric acid, beeswax, centella asiatica extract, and rosehip to nourish the skin. The remaining components of the cream include aqua, glycerin, disodium edta, glyceryl stearate, PEG‐100 stearate, hydrogenated polidecene, tridecth‐6, sodium polyacrylate, caprylyl glycol, glycerin caprylate, dipropylene glycol, sodium benzoate, tocopherol, beta‐sitosterol, squalene, caprylic/capric triglyceride, and hydroxymethoxyphenyl decanone.

Fisiocrem (Productos para preparar Músculos | Fisiocrem) contains natural extracts such as arnica ( *Arnica Montana* ) flower extract, calendula ( *Calendula Officinalis* ) extract, hypericum ( *Hypericum Perforatum* ) extract, *Melaleuca alternifolia* leaf extract, canola oil, and menthol. The remaining components of the cream include aqua, pentylene glycol, glyceryl stearate, PEG‐100 stearate, carbomer, acrylates/C 10–30 alkyl acrylate crosspolymer, and sodium hydroxide.

### 2.3 Triangular Methodology for Sensorial Testing

**FIGURE 1:**
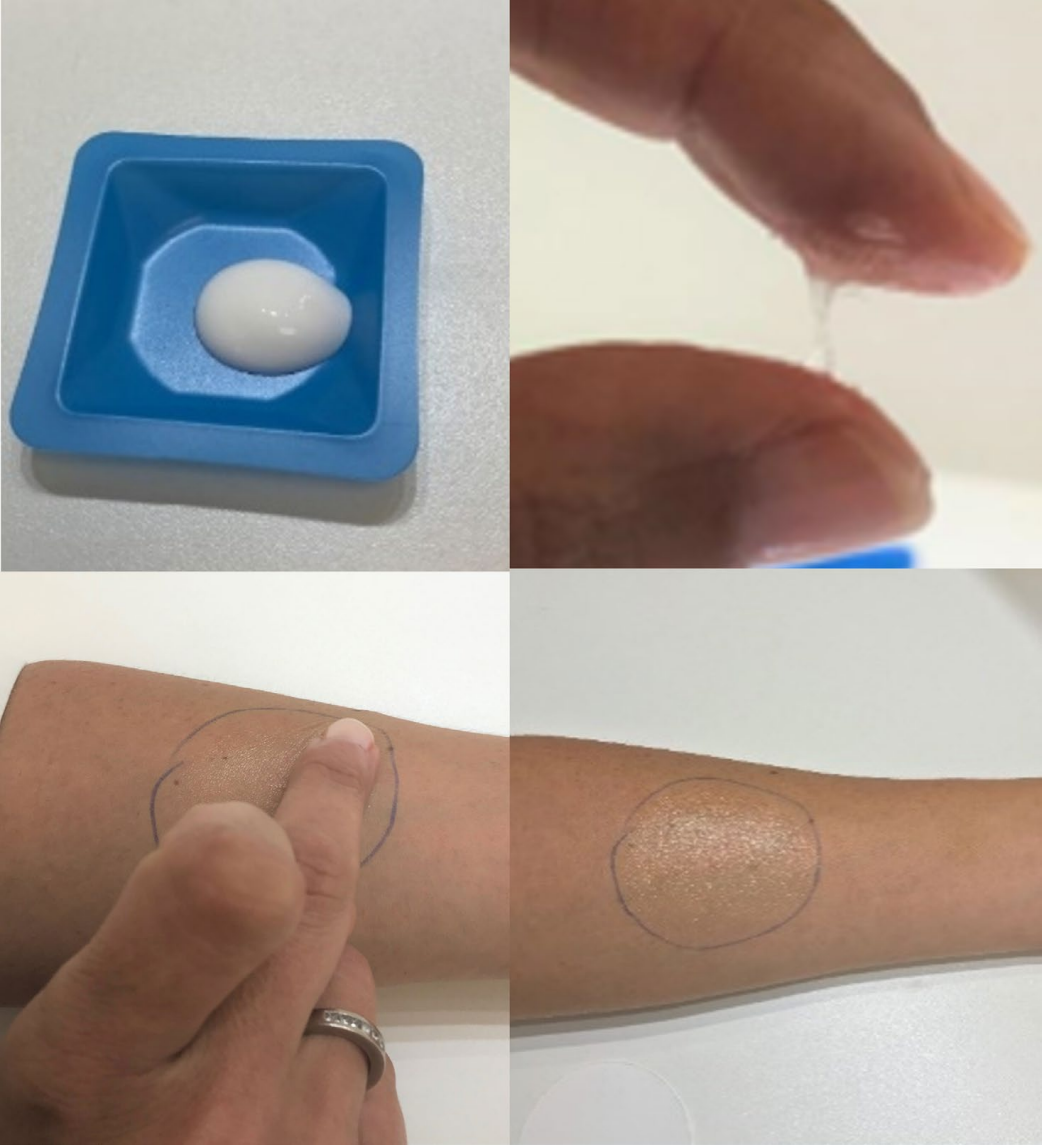
Recreation of a sensory session with one of the products to be assessed. Above, container (weight dish) used by each panelist with a sample of product. Below, example of application of the product on a delimited area of the forearm (5 cm diameter).

A triangle methodology was used for the sensory evaluation of the products [23]. For the presentation of the samples, a sterilized weight dish was used. All samples were blinded, and the order was randomized for each panelist. The amount of product applied as standard was the equivalent of one grain of rice on a circular surface (created by the use of a flexible template) of approximately 5 cm diameter, on the inner side of the forearm until its complete absorption [22], as shown in Figure 1. A consistent light source for each expert was used during the evaluation of shine properties. All experts received the same amount of light on the arm. The angle of light and the distance from the test site were the same for each panelist.

### 2.4 Variables and Assessment Tools

The current methodology is based on a qualitative and a quantitative approach.

#### 2.4.1 Qualitative Assessment: Preference Test for Overall Product Rating and Cosmetic Qualities of the Products

The study included a questionnaire asking questions about the overall assessment of the products to be compared (Physiorelax and Fisiocrem), as well as an assessment of different cosmetic qualities of the products. The questionnaires were separate for each product.

The overall rating of each product was carried out using a scale with seven possible answers: A, “I like it very much”; B, “I quite like it”; C, “I like it a little”; D, “I neither like it nor dislike it”; E, “I don't quite like it”; F, “I don't like it very much”; and G, “I don't like it at all”.

The cosmetic qualities of the two products were assessed by 19 items (see Table S1), which asked about the degree of agreement on different qualities related to the application of the products, texture, smell, color, feel, product duration or absorption, among other aspects. Each item was evaluated on a scale of four possible responses: A, “Completely agree”; B, “Slightly agree”; C, “Slightly disagree”; and D, “Completely disagree”.

In addition, a qualitative question was presented to the panelists in which they could indicate further observations in relation to the products, the sensations generated, and/or their effects.

#### 2.4.2 Quantitative Assessment: Organoleptic Test for Perceptual Qualities of the Application of the Products

The evaluation of the perceptual characteristics of the application of the products was assessed by a questionnaire including 20 items evaluating four dimensions: afterfeel (physical and kinaesthetic attributes of the skin surface after the use of the product to measure the residues left by the product. 8 items: amount of residue after 10 s, amount of residue, slipperiness after 10 s, slipperiness, sticky after 10 s, sticky, gloss after 10 s, and gloss), appearance (attributes of a product measured based on the sense of sight which may include, but are not limited to, the rheological, optical attributes of a product measured when handled between the fingers. 3 items: integrity of shape, integrity of shape after 10 s, and gloss), pick up (rheological attributes of a product measured when handled between the fingers. 4 items: firmness, stickiness, cohesiveness, and amount of peaking), and rub out (physical and rheological attributes of a product on the skin measured when the product is spread on the skin until it is absorbed in addition to any kinaesthetic sensations that may occur. 5 items: absorbency, grease, thickness, spreadability, and wetness). The items were rated on a scale of 0–100, where 0 was absence of the property and 100 was maximum presence of the assessed property.

### 2.5 Statistical Analysis

For the overall assessment of the products, the scale of seven possible initial responses was summarized into three categories of panelists' rankings: “They do like the product” (answers A, B, and C); “They do not like the product” (answers E, F, and G); and “They are indifferent to the product” (answer D).

For the assessment of cosmetic qualities, the scale of four possible initial responses was summarized into two rating categories: “Agree with the item statement” (responses A and B); and “Disagree with the item statement” (responses C and D).

All qualitative results are shown as the percentage of panelists placed in one or the other response category. To assess the difference between proportions, the chi‐square test has been used.

For the assessment of the physical properties related to the topical application of the products (and, therefore, their applicability as massage products), a radial graph (or network graph) was generated to visually compare the quantitative values (on a scale from 0 to 100) of the different aspects of the application for each of the products assessed.

## 3 Results

### 3.1 Preference Test Results for Overall Product Rating and Cosmetic Qualities of the Products

**FIGURE 2:**
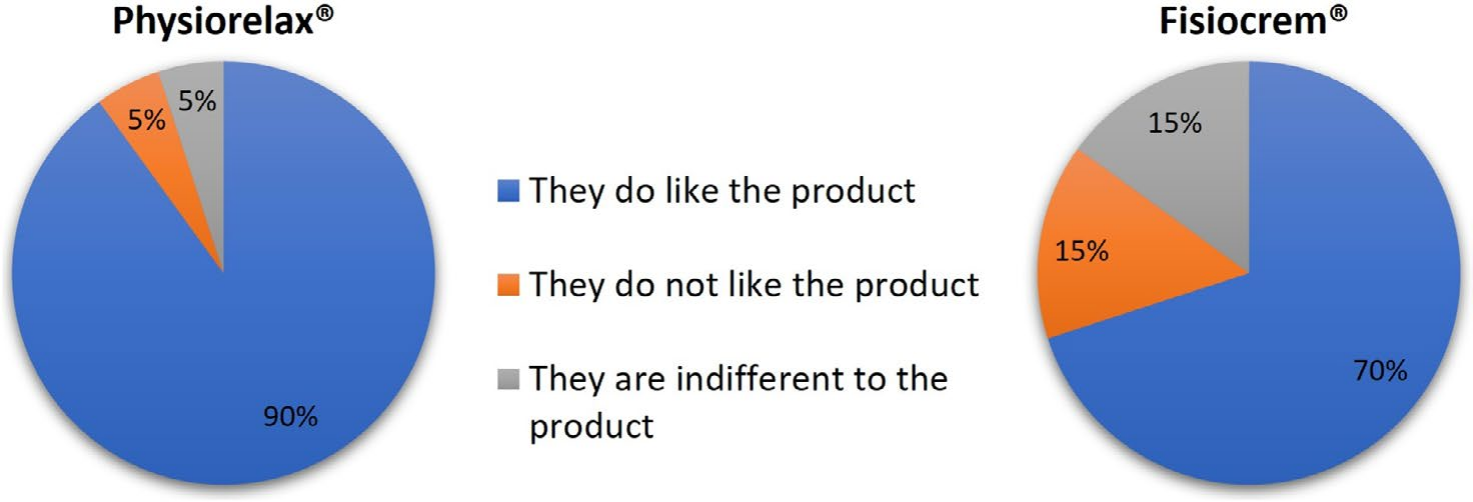
Overall rating of products: Percentage of panelists responding about their overall preference for products. The difference between the two creams in terms of product preference was statistically significant (*p* = 0.002).

The overall assessment of the products in terms of whether the panelists liked or disliked them is shown in Figure 2. While 90% of panelists liked Physiorelax, 70% liked Fisiocrem. An additional 10% of panelists indicated that they disliked Fisiocrem over Physiorelax (chi‐square = 12.5, *p* = 0.002) (Figure S1 shows the overall rating of each panelist for the two products considering the seven original response possibilities).

**FIGURE 3:**
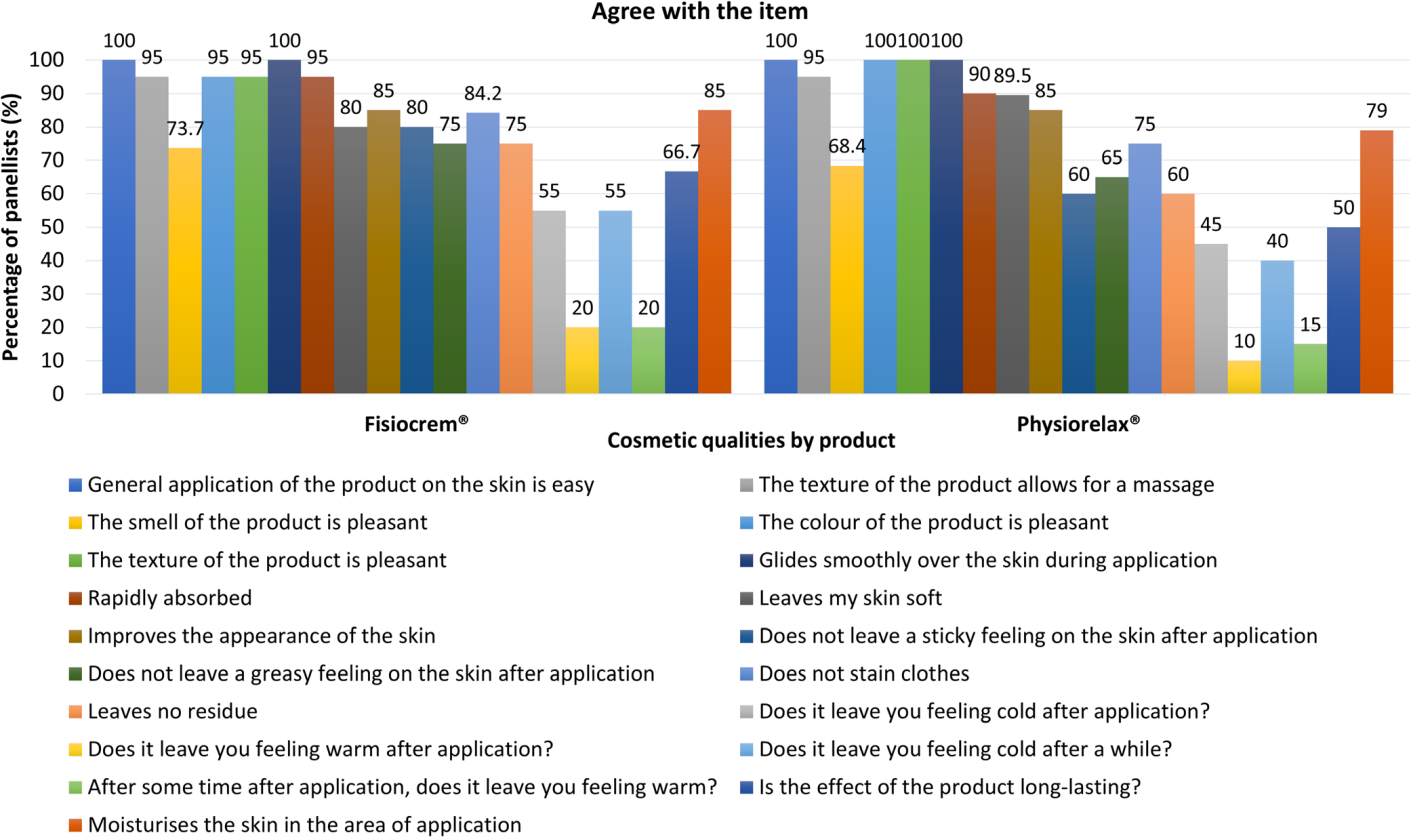
Level of agreement between panelists about the cosmetic qualities of the products. Percentages calculated based on the final *N* for each item.

The level of agreement of the panelists with each individual item related to the cosmetic qualities of the products is shown in Table 1 and Figure 3. In both cases, 16 out of the 19 evaluated organoleptic aspects showed a percentage of agreement between panelists ≥ 50%. Both products showed a similar overall response profile, although in the case of Fisiocrem a slightly higher percentage of panelists agreed that the cold sensation lasts longer, leaving a less sticky/greasy feeling on the skin after application, and less residue. In the case of Physiorelax, a slightly higher percentage of panelists considered that the product left the skin feeling softer after application, and all experts (100%) agreed that the color and texture of the product were pleasant. All panelists (100%) agreed that both products were easy to apply and glided smoothly over the skin during application.

**TABLE 1:** Level of agreement between panelists about the cosmetic qualities of the products.

Item	Fisiocrem		Physiorelax	
	Agree	Disagree	Agree	Disagree
General application of the product on the skin is easy	100	0	100	0
The texture of the product allows for a massage	95	5	95	5
The smell of the product is pleasant	73.7	26.3	68.4	31.6
The color of the product is pleasant	95	5	100	0
The texture of the product is pleasant	95	5	100	0
Glides smoothly over the skin during application	100	0	100	0
Rapidly absorbed	95	5	90	10
Leaves my skin soft	80	20	89.5	10.5
Improves the appearance of the skin	85	15	85	15
Does not leave a sticky feeling on the skin after application	80	20	60	40
Does not leave a greasy feeling on the skin after application	75	25	65	35
Does not stain clothes	84.2	15.8	75	25
Leaves no residue	75	25	60	40
Does it leave you feeling cold after application?	55	45	45	55
Does it leave you feeling warm after application?	20	80	10	90
Does it leave you feeling cold after a while?	55	45	40	60
After some time after application, does it leave you feeling warm?	20	80	15	85
Is the effect of the product long‐lasting?	66.7	33.3	50	50
Moisturizes the skin in the area of application	85	15	79	21

### 3.2 Qualitative Observation of Responses to the Preference Test

The qualitative observations of the two products can be found in Figure S2.

### 3.3 Organoleptic Test Results for Physical Qualities of Application of the Products

**FIGURE 4:**
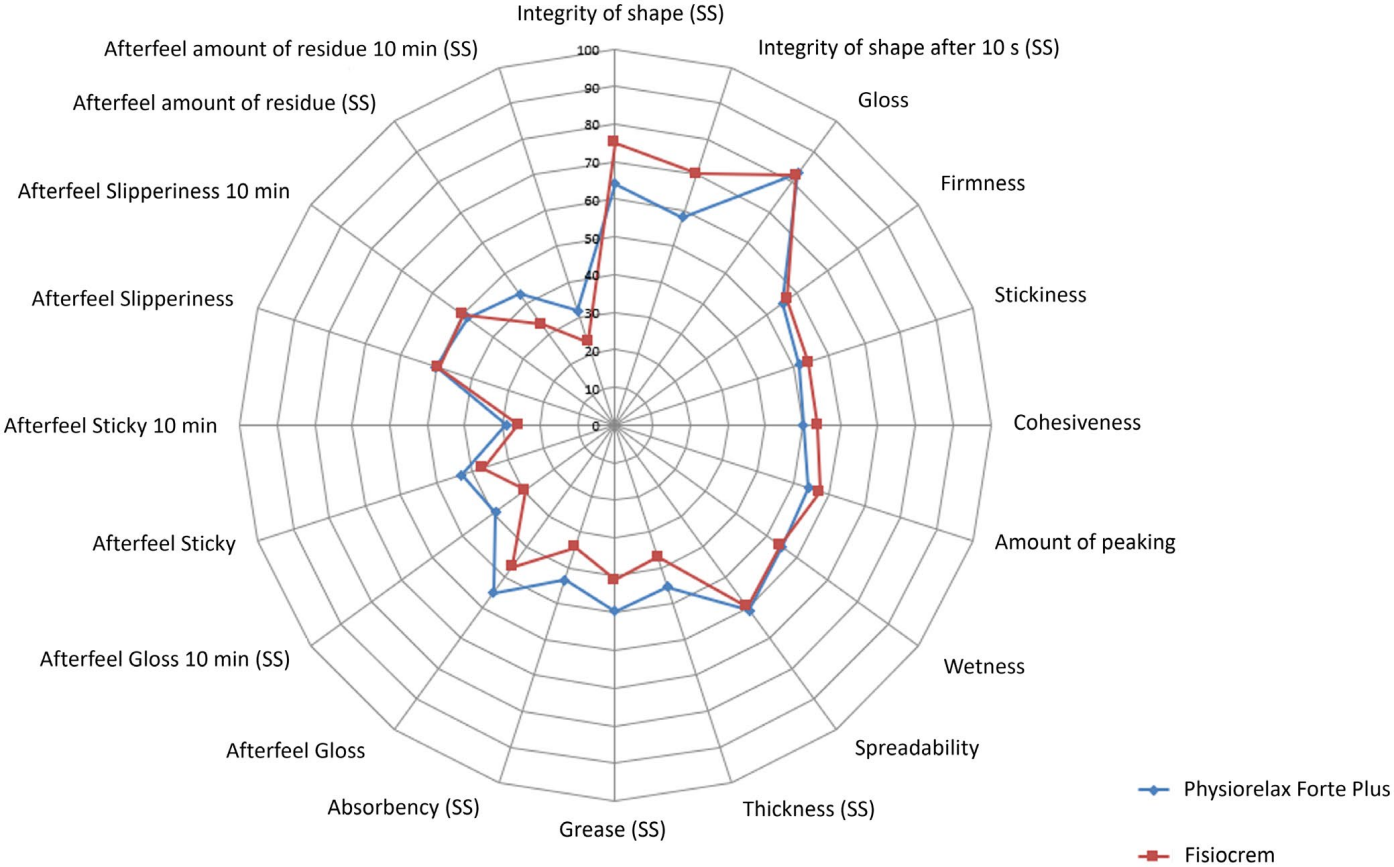
Radial graph comparing the physical application properties of Physiorelax versus Fisiocrem. Scale of values between 0 and 100. Dimensions assessed: Afterfeel (8 variables), appearance (3 variables), pick up (4 variables), and rubbing (5 variables). SS, Significant statistical difference.

Figure 4 compares the assessment of the four dimensions related to the topical application of the two products (afterfeel, appearance, pick up, and rubbing) on a scale from 0 to 100. Overall, while Physiorelax scored higher in aspects related to afterfeel and rubbing, it scored slightly lower in pick up. Fisiocrem showed higher values in integrity of shape despite both products being above 60. The lowest scores (< 35) for the two products were found for the item afterfeel amount of residue after 10 min.

## 4 Discussion

Not only for athletic or generally active persons, but also for daily health care prevention or muscle relief at any adult age and for any condition, regular massages can be a necessity, as they can be an instrument to help the body recover and maintain health [24]. Benefits of regular massages are multiple, both physically and mentally [25, 26], overall improving the quality of life. It has been reported that regular therapy massage reduces levels of cortisol while increasing levels of dopamine and serotonin [27].

Currently, within personal musculoskeletal health care and pain relief, numerous possibilities exist, and natural products, including herbal and essence therapies, play an increasingly main role. We present for the first time a galenic study by an expert panel methodology in sensory analysis of topical products evaluating the cosmetic and physical application properties of a natural topical cream for muscle massage, Physiorelax (Forte Plus Cream), as well as the overall rating in terms of preference of the product compared to Fisiocrem.

The results display a similar sensory profile between the two products (e.g., good shape integrity and firmness). Rating these physical aspects of the products helps to get an idea of the applicability of the products as an optimal massage cream/gel for use by professionals (e.g., physiotherapists). However, in global terms, our results point out that, under blinded conditions for the products' samples, Physiorelax cream is liked as a whole by significantly more panelists than those panelists who liked Fisiocrem. The latter product left 10% more panelists indifferent to it. The pleasant texture of Physiorelax and the fact that it leaves the skin soft seem to be two important elements when deciding on a topical massage product. The experts have rated them higher for Physiorelax than for Fisiocrem. In addition, the absorption time in the average and the slightly greater greasy feeling above Fisiocrem powers Physiorelax's bonanza, since physiotherapists and sports users of this type of product are looking for a not too long but sufficient absorption time to allow the correct manual therapy without having to reapply the product and a higher sliding capacity (i.e., a greasier product).

One of the fundamental differences between the two products is the increase of natural substances included in the galenic composition of Physiorelax compared to Fisiocrem, some of which are intended for the health care professional who performs massage in his or her daily practice.

The extra components of Physiorelax are aimed in part at the health of the skin itself, both of the person who receives the product and of the musculoskeletal health professionals that apply it. Sunflower extract and beeswax have been described as a natural repairer of the skin barrier function [28, 29]. Interestingly, beeswax (as a natural substance) has been shown to alleviate symptoms associated with common cutaneous conditions (e.g., like dermatitis, psoriasis), so the use of Physiorelax seems appropriate even in the most sensitive skin [30, 31].

Among other extra components of Physiorelax, limonene and centella asiatica extract (the latter with moisturizing skin properties) act as antioxidants, which play a vital role in skincare as they combat free radicals, avoiding premature aging and skin damage [32, 33, 34]. Finally, rosehip is known for its multiple benefits for the skin, such as moisturizing and hydration [35, 36], helping boost collagen formation [36], reducing inflammation [37], and helping boost immunity [38], which makes it an ideal substance to be used on a regular basis, on any type of skin.

With the inclusion of ingredients related to skin care such as centella or rosehip, the formulation of Physiorelax also considers the hands care of the professional providing the massage, making it a very interesting option for muscle and ligament massage, as it considers the skin of both the person receiving and providing the massage.

In global terms, the differences in the ingredients of Physiorelax and Fisiocrem affect their cosmetic qualities in several ways. Physiorelax contains a combination of high‐quality plant extracts (arnica, *harpagophytum, hypericum* and calendula) and additional natural ingredients (e.g., mentha, menthol, sunflower extract, rosa moschata seed oil, glycine soja oil, beeswax and thermal water, among others). These ingredients not only contribute to the product's efficacy in musculoskeletal care but also improve skin health by providing moisturizing and repairing properties (antioxidant function, by reducing inflammation and increasing collagen formation preventing premature skin aging), helping restoration of the skin barrier function and relieving symptoms associated with common skin conditions such as dermatitis and psoriasis. This makes Physiorelax suitable even for sensitive skin.

Although panel studies have many advantages, such as the experience of the panelists in assessing the products, this study is not without limitations. First, we cannot deny the existence of a certain degree of panelist bias inherent in the design. To minimize this bias related to a certain degree of subjectivity, the samples were randomized and blinded. Second, the findings may not be generalizable to a larger population, precisely because of the expertise of the panelists in the assessment. However, the study sought to measure the organoleptic characteristics of the products through experts, so the panel approach would be appropriate.

In summary, for massages to be regular in health maintenance, the cosmetic and personal care industries must place special emphasis on the organoleptic characteristics of the topical products so that their use becomes routine. A solid knowledge of the sensorial and dermatological skin‐related properties has an important role in usage decisions. In this process, the figure of the massage therapist also comes into play in professional practice, whose sensory experience of the product is very important for massage application. Thus, the choice of the galenic formulation is, therefore, an important decision in the world of personal care products affecting the convenience toward product application [39]. The results derived from experts in the field facilitate the decision on the use of the products by health personnel involved in muscle and ligament massage. Current findings point to a good profile of Physiorelax in terms of massage product, respecting skin care, and a preference for its use by the expert panel over Fisiocrem. Although Fisiocrem also contains natural ingredients (e.g., arnica, calendula, and hypericum), the Physiorelax formulation includes additional components that may explain the variations in cosmetic qualities observed during the study, that not only benefit the user's skin, but also that of the massage practitioner, making the latter a complete option for musculoskeletal care and skin health. Future studies could be aimed at assessing the preference of use of the product by the general population and the health care professionals providing muscle and ligament massages.

## 5 Conclusion

The two topical evaluated products present similar physical sensory characteristics; however, a higher percentage of experts prefers Physiorelax as a whole and according to its cosmetic qualities. This fact, together with its good properties for muscle and ligament massage and the incorporation of specific products for the care of the skin, makes this product a very good option for regular musculoskeletal care.

## Conflicts of Interest

N.G.G., P.G.C., D.A.T., and S.P.L. are employers of Almirall S.A. J.B.N. has made a presentation on Physiorelax in a national convention organized by Almirall. M.G. has no conflicts of interest.

## Supporting information


Appendix S1

## Data Availability

The data that support the findings of this study are available from the corresponding author upon reasonable request.
