# Supplementary material for: Cosmetic and Application Qualities of the Cream Physiorelax Compared to Another Topical Cream for Musculoskeletal Health Care: An Organoleptic Expert Panel Approach
Source: J Cosmet Dermatol. 2025 Sep 22;24(10):e70368. doi: 10.1111/jocd.70368 (PMC12455026; doi:10.1111/jocd.70368)
Supplement: Supplementary file 1 — Appendix S1 [file JOCD-24-e70368-s001.docx]

SUPPLEMENTARY MATERIAL

**Methods**

**Table S1**. Items about cosmetic qualities for each product (Physiorelax® and Fisiocrem®).

| **COSMETIC QUALITIES** | | | | |
| --- | --- | --- | --- | --- |
| **Item** | A) Completely agree | B) Slightly agree | C) Slightly disagree | D) Completely disagree |
| **The general application of the product on the skin is easy** |  |  |  |  |
| **The texture of the product allows for a massage** |  |  |  |  |
| **The smell of the product is pleasant** |  |  |  |  |
| **The colour of the product is pleasant** |  |  |  |  |
| **The texture of the product is pleasant** |  |  |  |  |
| **Glides smoothly over the skin during application** |  |  |  |  |
| **Absorbs quickly** |  |  |  |  |
| **Leaves my skin feeling soft** |  |  |  |  |
| **Improves the appearance of the skin** |  |  |  |  |
| **Does not leave a sticky feeling on the skin after application** |  |  |  |  |
| **Does not leave a greasy feeling on the skin after application** |  |  |  |  |
| **Does not stain clothes** |  |  |  |  |
| **No residue** |  |  |  |  |
| **Does it leave a warm sensation after application?** |  |  |  |  |
| **Does it leave a cold sensation after application?** |  |  |  |  |
| **After some time after application, does it leave you feeling cold?** |  |  |  |  |
| **After some time after application, does it leave you feeling warm?** |  |  |  |  |
| **Is the effect of the product long lasting?*** |  |  |  |  |
| **Moisturises the skin in the area of application** |  |  |  |  |

***If yes* Approximately how long do you perceive the product to last (open field)?**

**Indicate other observations in relation to the product and its sensations/effects:**

**Results**

**Figure S1.** Overall product rating: percentage of panellists responding about their overall preference for the products, for each of the 7 categories of response to the preference questionnaire.


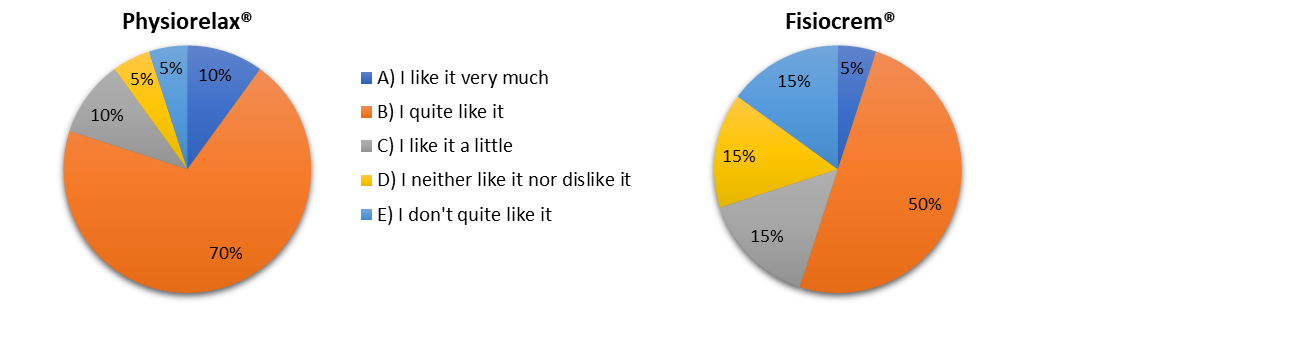


Responses F and G do not appear in the legend, as no panellist indicated them.

**Figure S2.** Qualitative comments from panellists adding some of the sensations perceived.


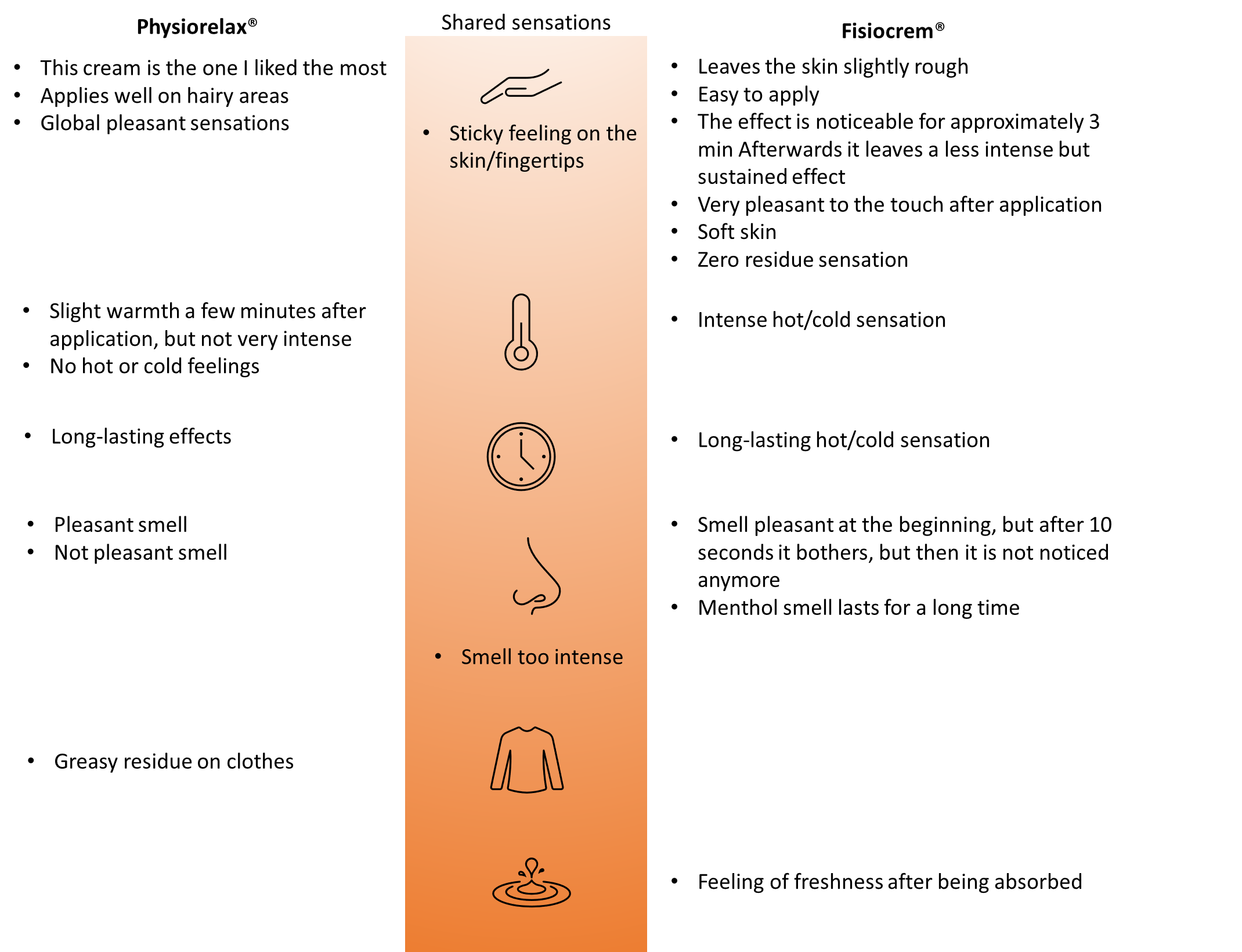


The coloured column represents the comments to some sensations common to both products.
